# Carrier-specific dynamics in 2H-MoTe_2_ observed by femtosecond soft x-ray absorption spectroscopy using an x-ray free-electron laser

**DOI:** 10.1063/4.0000048

**Published:** 2021-01-13

**Authors:** Alexander Britz, Andrew R. Attar, Xiang Zhang, Hung-Tzu Chang, Clara Nyby, Aravind Krishnamoorthy, Sang Han Park, Soonnam Kwon, Minseok Kim, Dennis Nordlund, Sami Sainio, Tony F. Heinz, Stephen R. Leone, Aaron M. Lindenberg, Aiichiro Nakano, Pulickel Ajayan, Priya Vashishta, David Fritz, Ming-Fu Lin, Uwe Bergmann

**Affiliations:** 1 Stanford PULSE Institute, SLAC National Accelerator Laboratory, Menlo Park, California 94025, USA; 2 Linac Coherent Light Source, SLAC National Accelerator Laboratory, Menlo Park, California 94025, USA; 3 SUNCAT Center for Interface Science and Catalysis, SLAC National Accelerator Laboratory, Menlo Park, California 94025, USA; 4 Department of Materials Science and NanoEngineering, Rice University, Houston, Texas 77005, USA; 5 Department of Chemistry, University of California, Berkeley, California 94720, USA; 6 Stanford Institute for Materials and Energy Sciences, SLAC National Accelerator Laboratory, Menlo Park, California 94025, USA; 7 Collaboratory for Advanced Computing and Simulations, University of Southern California, Los Angeles, California 90089, USA; 8 PAL-XFEL, Pohang Accelerator Laboratory, 80 Jigokro-127-beongil, Nam-gu, Pohang, Gyeongbuk 37673, South Korea; 9 Stanford Synchrotron Radiation Lightsource, SLAC National Accelerator Laboratory, Menlo Park, California 94025, USA; 10 Department of Applied Physics, Stanford University, Stanford, California 95305, USA; 11 Chemical Sciences Division, Lawrence Berkeley National Laboratory, Berkeley, California 94720, USA; 12 Department of Physics, University of California, Berkeley, California 94720, USA; 13 Department of Materials Science and Engineering, Stanford University, Stanford, California 94305, USA

## Abstract

Femtosecond carrier dynamics in layered 2H-MoTe_2_ semiconductor crystals have been investigated using soft x-ray transient absorption spectroscopy at the x-ray free-electron laser (XFEL) of the Pohang Accelerator Laboratory. Following above-bandgap optical excitation of 2H-MoTe_2_, the photoexcited hole distribution is directly probed via short-lived transitions from the Te 3*d*_5/2_ core level (M_5_-edge, 572–577 eV) to transiently unoccupied states in the valence band. The optically excited electrons are separately probed via the reduced absorption probability at the Te M_5_-edge involving partially occupied states of the conduction band. A 400 ± 110 fs delay is observed between this transient electron signal near the conduction band minimum compared to higher-lying states within the conduction band, which we assign to hot electron relaxation. Additionally, the transient absorption signals below and above the Te M_5_ edge, assigned to photoexcited holes and electrons, respectively, are observed to decay concomitantly on a 1–2 ps timescale, which is interpreted as electron–hole recombination. The present work provides a benchmark for applications of XFELs for soft x-ray absorption studies of carrier-specific dynamics in semiconductors, and future opportunities enabled by this method are discussed.

## INTRODUCTION

The optically excited carrier relaxation and structural dynamics of semiconductors govern their optoelectronic properties and functionality in emerging device applications.^1^ Femtosecond time-resolved x-ray spectroscopy and scattering are powerful techniques to track the electronic and structural dynamics of such materials in real time. With the recent development of ultrafast x-ray and extreme ultraviolet (XUV) sources using x-ray free electron lasers (XFELs) and high-harmonic generation (HHG), new opportunities are being explored for investigating charge-carrier dynamics in the condensed phase with carrier-, element-, and oxidation-state specificity.^2–5^ The ability to probe the valence electronic structure via localized core levels using x-ray/XUV absorption spectroscopy (XAS) has been exploited to capture electron and hole carrier relaxation dynamics separately in bulk semiconductors^6–12^ and to measure layer-specific dynamics within multi-component heterojunctions.^13^ These early successes have been led primarily by applications of table-top HHG sources for XUV transient absorption and reflection spectroscopy.^3^

With the recent emergence of XFELs, which produce femtosecond x-ray pulses with ∼6–8 orders of magnitude greater spectral brightness per pulse compared to HHG,^14^ a new horizon is coming into view for ultrafast core-level spectroscopy of semiconductors. In addition to the potential for detecting weak signals using a high XFEL photon flux, the small spatial scale (∼1–10's *μ*m)^15,16^ of the focused x-ray pulses, and the much longer penetration depths of higher-energy x-rays allow for smaller and thicker semiconductor samples, respectively, to be investigated. Furthermore, the small spin–orbit energy splittings of elemental core levels within the XUV range, which are often comparable to the bandgap of relevant semiconductors (1–3 eV), lead to overlapping spectral features that frequently complicate the interpretation of hole and electron dynamics in XUV spectroscopy.^6–8,10,12,17^ Although deconvolution of the spin–orbit split transitions has been possible in some examples,^6,10,17^ the overlapping spin–orbit features can be completely eliminated by using the higher-energy x-rays from XFELs at deeper core-level edges that are well-separated compared to the bandgap energies.^18^ In the present work, we apply femtosecond optical-pump, x-ray-probe spectroscopy at the Te M_5_-edge (572–577 eV) using an XFEL to capture the carrier-specific dynamics of a prototypical layered semiconductor, 2H-MoTe_2_. Our study provides a benchmark for XFEL-based soft x-ray femtosecond transient absorption spectroscopy of semiconductor materials.

MoTe_2_ is a transition metal dichalcogenide (TMDC) within the class of layered materials, like graphite, that are composed of two-dimensional sheets (monolayers) bound by weak van der Waals forces. In contrast to graphite and its corresponding monolayer form, graphene, which are both semimetallic, MoTe_2_ is stable in the bulk and as a monolayer in both a semimetallic phase (1T′) and semiconductor phase (2H).^19,20^ The 2H-phase MoTe_2_ has a bandgap of ∼0.9 eV in the bulk and 1.1 eV in the monolayer form,^21^ which are similar to that of silicon (1.1 eV). Various applications based on both monolayer and multilayer MoTe_2_ have been investigated, including field-effect-transistors,^22,23^ photonic logic gates,^24^ and phase-change devices.^25^ The advancement of next-generation optoelectronics based on layered TMDC materials relies on a detailed understanding of the relaxation and transport of the carriers.^24,26^ Therefore, recent studies using ultrafast optical and THz absorption spectroscopies have examined carrier lifetimes in 2H-MoTe_2_.^27–29^ However, these measurements lack both carrier-specificity and sensitivity to hot electron and hole dynamics prior to electron–hole recombination or trapping.

Time-resolved XAS can distinguish electron and hole dynamics in semiconductors, including intraband carrier thermalization and cooling.^7,9,12^ To achieve this, an optical pump pulse first excites electrons into the conduction band (CB) and creates holes in the valence band (VB); a temporally delayed x-ray pulse then probes the changes in transitions of core-level electrons into the unoccupied states of the VB and CB. The effects of increased core → VB transitions due to the presence of photoexcited holes and decreased core → CB transitions due to excited electrons are collectively referred to as state-filling effects.^6,10,17^ In a recent work, using XUV transient absorption spectroscopy on 2H-MoTe_2_, we captured the hot hole scattering dynamics within the VB, the subsequent electron–hole recombination, and coherent lattice dynamics in 2H-MoTe_2_ induced by strong electron–phonon coupling.^12^ The 1.5 eV spin–orbit splitting of the Te N_5_ and N_4_ edges, which is comparable to the 2H-MoTe_2_ bandgap energy (0.9 eV), led to overlapping spectral features, particularly between the hole signal at the N_4_ edge and the electron state-filling signal at the N_5_ edge. Unfortunately, this overlap precluded a detailed analysis of the electron thermalization and cooling dynamics.

In the present work, we extend the method established by XUV transient absorption to the x-ray regime at the Te M_5_ edge of 2H-MoTe_2_ using an XFEL. We achieve a noise level of ∼0.05% in measurements of the x-ray transmission, which surpasses the performance of XUV measurements on the same material and is crucial due to an order-of-magnitude smaller edge jump in absorption at the M_5_ edge compared to the N_5,4_ edge in the XUV. At the Te M_5_ edge, which is spectrally isolated by >10 eV from neighboring core-level edges, we show that the XAS closely maps the unoccupied density of states (DOS) of the CB in 2H-MoTe_2_. In this time-resolved optical-pump, XAS-probe experiment, we identify a signature of hot electron cooling within the CB, which was not observed in the recent XUV study due to overlapping state-filling signals from the N_5_ and N_4_ edges. In addition, we successfully capture the separate photoexcited hole signal and trace the electron–hole recombination dynamics of 2H-MoTe_2_.

## EXPERIMENTAL METHODS AND CALCULATIONS

### Sample preparation and static XAS measurements using synchrotron radiation

The static XAS of both the 1T′ and 2H phases of MoTe_2_ are measured in the present work, while pump–probe measurements are carried out only on 2H-phase MoTe_2_. Samples of 2H- and 1T′-MoTe_2_ are synthesized via chemical vapor deposition (CVD) and directly deposited on 100 nm thick Si_3_N_4_ substrates of 2 × 2 mm^2^ lateral size. This results in a homogenous thin-film polycrystalline sample for both phases. The structural phase of the samples is confirmed by their Raman spectra (see the supplementary material for more details).^20^ The static XAS measurements of the 2H and 1T′ phases of MoTe_2_ were initially carried out at Beamline 8–2 of the Stanford Synchrotron Radiation Lightsource (SSRL) synchrotron tuned to the Mo M_3_ and Te M_5,4_ edges. Further information about soft x-ray absorption spectroscopy at the SSRL can be found in the literature.^30^ Several experimental approaches were considered for soft x-ray absorption measurements. In contrast to 3*d* transition metal L edges,^31^ a total or partial fluorescence yield measurement of the Mo and Te M edges is challenging because of their extremely low fluorescence yields of 0.3% and 0.1%, respectively.^32^ Thus, either a total electron yield (TEY) measurement or a transmission measurement would be more viable. Since the final goal is to incorporate these XAS measurements into pump–probe studies and the TEY measurement is more prone to artifacts induced by the optical pump laser,^33^ we focus in this work on the transmission measurement. To optimize the signal-to-noise ratio, the MoTe_2_ layer thickness during sample synthesis is tailored to obtain approximately one absorption length of the x-rays at the M_5_ edge. The resulting 100 nm MoTe_2_ layer absorbs ∼20% of incident x-rays at energies just below the Te M_5,4_ absorption edges and ∼54% above, which results in a change in the transmission by the absorption edge jump of ∼34%.^18^ However, we note that the change in transmission at the resonant M_5_ pre-edge—where the carrier dynamics are probed in the time-resolved measurements—is only 2%.

### Femtosecond time-resolved XAS using XFEL pulses

**FIG. 1.**
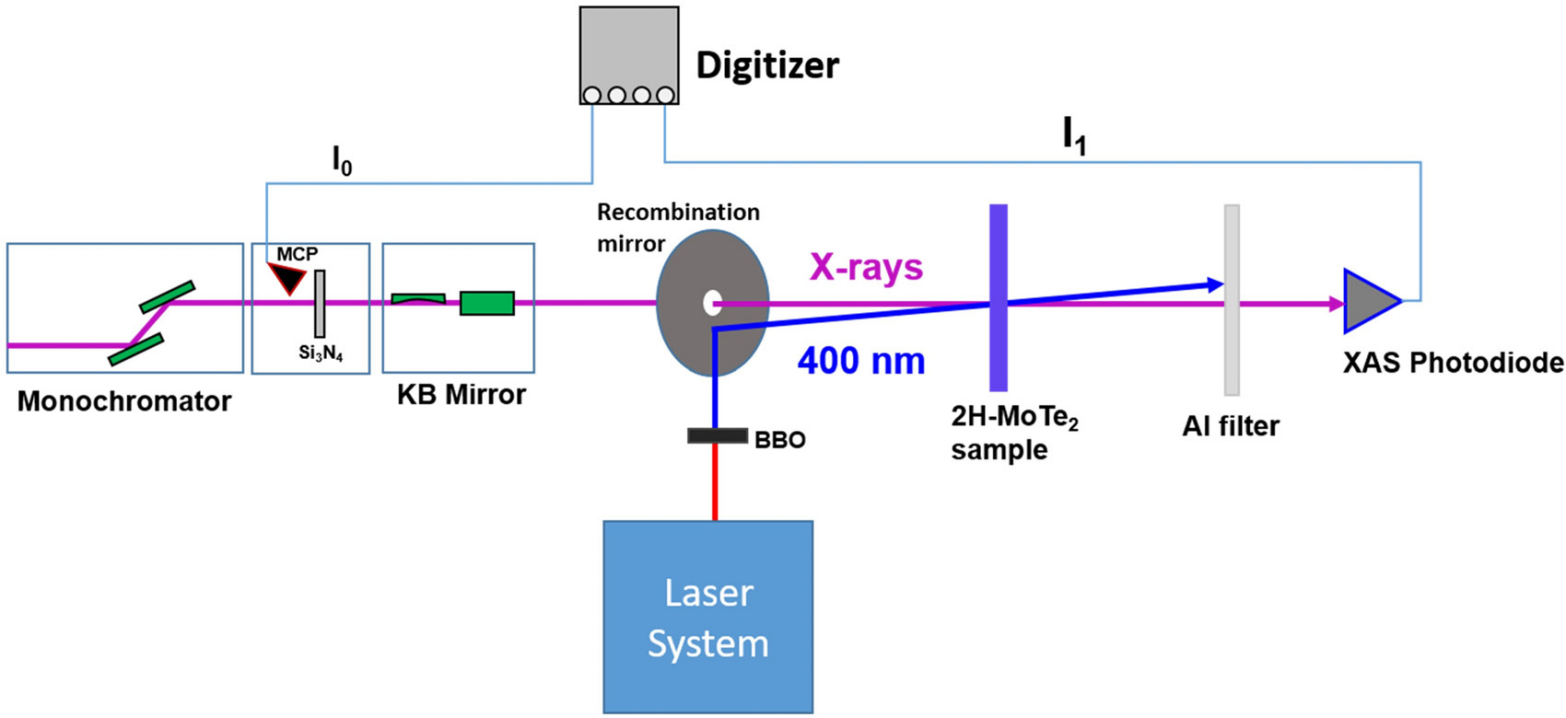
Schematic of the experimental setup at PAL-XFEL (not to scale). The incident x-ray intensity (I_0_) of each pulse is measured using the ejected photoelectrons from an Si_3_N_4_ membrane detected with an MCP detector. The transmitted x-ray intensity through the sample (I_1_) is measured with a photodiode.

The femtosecond time-resolved XAS measurements on 2H-phase MoTe_2_ were performed at the Soft X-ray Scattering and Spectroscopy (SSS) beamline of the Pohang Accelerator Laboratory (PAL)-XFEL. A simplified layout of the experiment is shown in Fig. 1; a detailed description of the soft x-ray beamline at PAL-XFEL can be found in the literature.^34^ In brief, the x-rays are monochromatized (to a bandwidth of 0.1 eV) and focused onto the sample with a Kirkpatrik–Baez (KB) mirror system, resulting in an x-ray beam diameter of 110 *μ*m × 80 *μ*m at the sample position, thus much smaller than the lateral extent of the thin-film MoTe_2_ sample. The monochromatized XFEL energy per pulse without further attenuation is measured as ∼4 *μ*J at 575 eV. In order to prevent x-ray-induced changes to the sample, including damage, the x-ray pulse energy is reduced by a series of Al filters (total thickness of 1.6 *μ*m) before the sample, resulting in an average pulse energy incident on the sample of approximately 0.24 *μ*J at the Te M_5_ pre-edge. For an XFEL pulse duration of ∼50 fs, this corresponds to a peak intensity of ∼5 × 10^10^ W/cm^2^. The relative number of photons/pulse (I_0_) of each incident x-ray pulse is measured using the ejected photoelectrons from a 150 nm thick Si_3_N_4_ membrane detected with an MCP detector. The relative x-ray photons/pulse transmitted through the sample (I_1_) is measured with a photodiode. Both detector signals are acquired with a 14-bit analog-to-digital converter (digitizer) and the integrated areas under the pulses are saved as I_0_ and I_1_.

In the pump arm, the output of a commercial Ti:sapphire amplifier is frequency-doubled to generate 400 nm pulses with ∼50 fs pulse duration. The 400 nm (3.1 eV) pump excitation is chosen to produce hot carriers in 2H-MoTe_2_ (bandgap of ∼0.9 eV), with a large energy contrast between hot carriers and cooled, band-edge carriers. The 400 nm pump beam is focused by a lens and reflected by an annular mirror onto the sample at an angle of ∼1° with respect to the x-rays. The laser spot size on the sample is 230 *μ*m (full width at half maximum, FWHM), much larger than the x-ray spot size. For a typical pump pulse energy of 34 *μ*J, the resulting excited carrier density is estimated to be 1.6 × 10^21^ carriers/cm^3^ (see the supplementary material for details on the optical absorption at 400 nm). The x-ray transmission photodiode (I_1_) is shielded from the transmitted and scattered 400 nm pump light with a free-standing 200 nm thick Al filter. The temporal pump–probe overlap is measured via the x-ray-induced change of 400 nm reflectivity with a single-crystal YAG sample. This measurement was carried out at least twice per 12-h shift and the drift in the centroid of the optical-x-ray temporal overlap was confirmed to be <100 fs on the timescale of a 6- to 12 h measurement. The pump–probe instrument response time is determined to be ∼200 fs, as described below.

## ELECTRONIC STRUCTURE CALCULATIONS

Density functional theory (DFT) with the projector augmented wave (PAW) method^35^ implemented in the Vienna *Ab initio* Simulation Package (VASP)^36,37^ was used to compute the density of states (DOS) for bulk 2H- and 1T′-phases of MoTe_2_ crystals without photoexcitation. Exchange and correlation effects were calculated using the Perdew–Burke–Ernzerhof (PBE) form of the Generalized Gradient Approximation (GGA). Wave functions were constructed using a plane wave basis set with components up to kinetic energy of 400 eV and the reciprocal space was sampled using a 3 × 3 × 3 Γ-centered mesh with a 0.05 eV Gaussian smearing of orbital occupancies. DFT simulations of bulk 2H (1T′) MoTe_2_ crystals were performed on 120-atom supercells measuring 17.58 Å × 12.19 Å × 13.97 Å (17.48 Å × 12.67 Å × 15.43 Å) along the *a*-, *b*-, and *c-*directions, respectively. Calculations were performed until each self-consistency cycle converged in energy to within $10^{-7}$ eV/atom and forces on ions are under $10^{-4}$ eV/Å.

The calculation of core-level absorption spectra at the Te M_5_ edge was accomplished with DFT and Bethe–Salpeter equation (BSE) calculations using Quantum ESPRESSO and the Obtaining Core-level Excitations using *Ab initio* methods and the NIST BSE solver (OCEAN) software package.^38–41^ The DFT-BSE calculation was conducted using norm-conserving scalar-relativistic PBE pseudopotentials with nonlinear core correction under GGA and a 6 × 6 × 1 k-point meshgrid.^42–45^ In the calculation, the number of bands was set to 40 and the dielectric constant was set to 12.9. Convergence was achieved with an energy cutoff of 80 Ryd and a cutoff radius of 4 Bohr. A lifetime broadening of 0.1 eV was assumed in the BSE calculation.

## RESULTS AND DISCUSSION

### X-ray absorption spectroscopy of 2H- and 1T′-phases of MoTe_2_

**FIG. 2.**
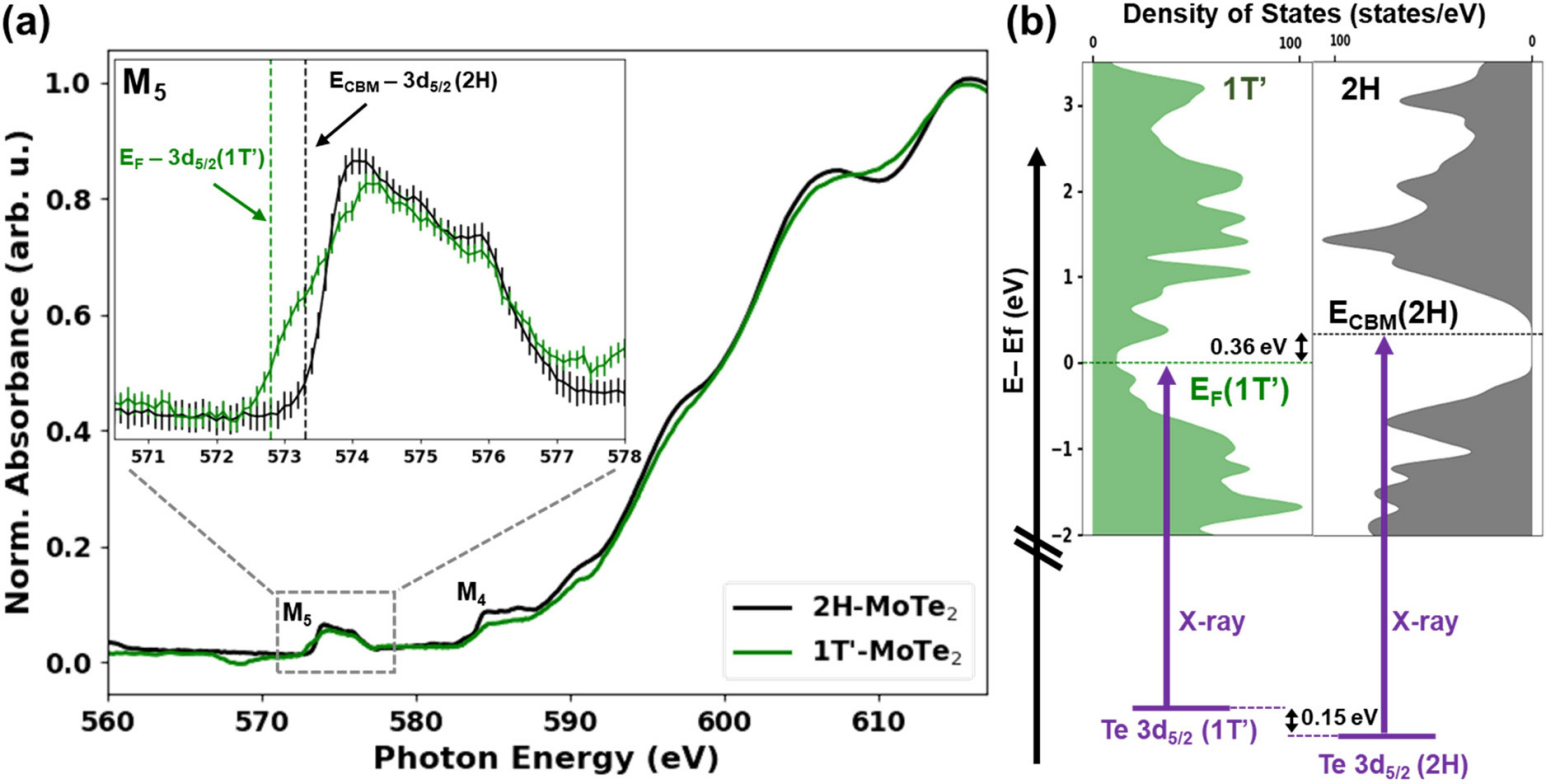
(a) Normalized Te M_5,4_-edge x-ray absorption spectrum of 2H- and 1T′-phases of MoTe_2_. Note the dip in absorption at ∼568 eV in the 1T′-MoTe_2_ spectrum is caused by an imperfect I_0_ normalization during refill of the synchrotron ring during that measurement. The inset shows an expanded plot of the Te M_5_ pre-edge feature. The green vertical dashed line denotes the energy of the Fermi level, E_F_, in 1T′-MoTe_2_ relative to the Te 3d_5/2_ core level of the 1T′ phase. The black vertical dashed line denotes the energy of the CB minimum, E_CBM_, in 2H-MoTe_2_ relative to the corresponding Te 3d_5/2_ core level of the 2H phase. (b) Calculated total DOS of bulk 2H- and 1T′-MoTe_2_. The dashed horizontal lines mark the calculated energies of E_F_(1T′) and E_CBM_(2H) with an energy scale set relative to the Fermi level of 1T′-MoTe_2_. The vertical purple arrows represent the onset of core-level transitions at the Te M_5_ edge of both phases, corresponding to the vertical dashed lines in (a). As the x-ray energy is increased above each onset, carriers are promoted to higher-energy states above E_F_(1T′) and E_CBM_(2H). Note the slight difference (0.15 eV) in the Te 3d_5/2_ core-level ionization potential in the 1T′-phase compared to the 2H-phase MoTe_2_ (see the supplementary material for more details), which gives a total difference of 0.51 eV in the expected XAS onsets of the two phases.

The static XAS of the 2H- and 1T′-phases of MoTe_2_ thin-film samples at the Te M_5,4_ edges acquired at SSRL, are shown in Fig. 2. The spectra cover the distinct M_5_ and M_4_ pre-edge features at 573 eV and 584 eV, respectively. The M_5_ and M_4_ pre-edge transitions correspond to the promotion of Te 3*d*_5/2_ and Te 3*d*_3/2_ core electrons, respectively, to the CB of semiconducting 2H-MoTe_2_ and to unoccupied states above the Fermi-level in 1T′-MoTe_2_, as depicted schematically in Fig. 2(b). The broad, atomic absorption to the continuum has its onset at higher energies, starting around the same energy as the M_4_-edge feature. This delayed onset is due to the centrifugal barrier.^46^ Note the dip in absorption at ∼568 eV in the 1T′-MoTe_2_ spectrum is caused by an imperfect I_0_ normalization during refill of the synchrotron ring during that measurement.

The inset of Fig. 2(a) shows in more detail the Te M_5_ pre-edge features of both structural phases of MoTe_2_. In the semimetallic 1T′-MoTe_2_, the Te M_5_ pre-edge onset is red-shifted compared to that of the semiconducting 2H-MoTe_2_. This is consistent with the bandgap collapse in the 1T′ phase compared to the 2H phase. To quantify this effect, we perform DFT calculations of the bulk DOS in both phases and extract the energy of the Fermi level, E_F_, in the case of bulk 1T′-MoTe_2_ and of the CB minimum, E_CBM_, in the case of bulk 2H-MoTe_2_, relative to the vacuum. The corresponding DOS distributions are plotted in Fig. 2(b) and the energy difference between E_CBM_(2H) and E_F_(1T′) is 0.36 eV. In the inset of Fig. 2(a), the vertical green dashed line denotes the energy difference between the calculated E_F_(1T′) and the measured core-level ionization potential of Te 3*d*_5/2_ (1T′), i.e., the energy of the transition represented by the purple vertical arrow on the left side of Fig. 2(b). The black vertical dashed line marks the energy difference between the calculated E_CBM_(2H) and the measured core-level ionization potential of Te 3*d*_5/2_ (2H), i.e., the energy of the transition represented by the purple vertical arrow on the right side of Fig. 2(b). Note that the Te 3*d*_5/2_ ionization potentials of the two phases are extracted directly from XPS measurements (see the supplementary material, Fig. S2), giving the 0.15 eV difference noted in Fig. 2(b), whereas the energy difference between E_F_(1T′) and E_CBM_(2H) of 0.36 eV comes from the DFT calculations. The overall expected red shift of the 1T′-MoTe_2_ M_5_ pre-edge onset relative to that of 2H-MoTe_2_ is therefore 0.15 eV + 0.36 eV = 0.51 eV, which is in good agreement with the experimental XAS. In both phases, although the M_5_ pre-edge transitions of Te(3*d*_5/2_) → Te(5*p*) character are dipole allowed, the absorption change (i.e., the edge jump) is weak at these resonances. As seen in Fig. 2(a), the pre-edge features are ∼20 times weaker than the continuum resonance of the Te M_5,4_ edge. For a 100 nm thick sample, the x-ray transmission only changes by ∼2% at the M_5_ pre-edge. The clear distinction between the 2H- and 1T′-phase Te M_5_-edge absorption illustrates the sensitivity of XAS to modifications in the valence electronic structure.

**FIG. 3.**
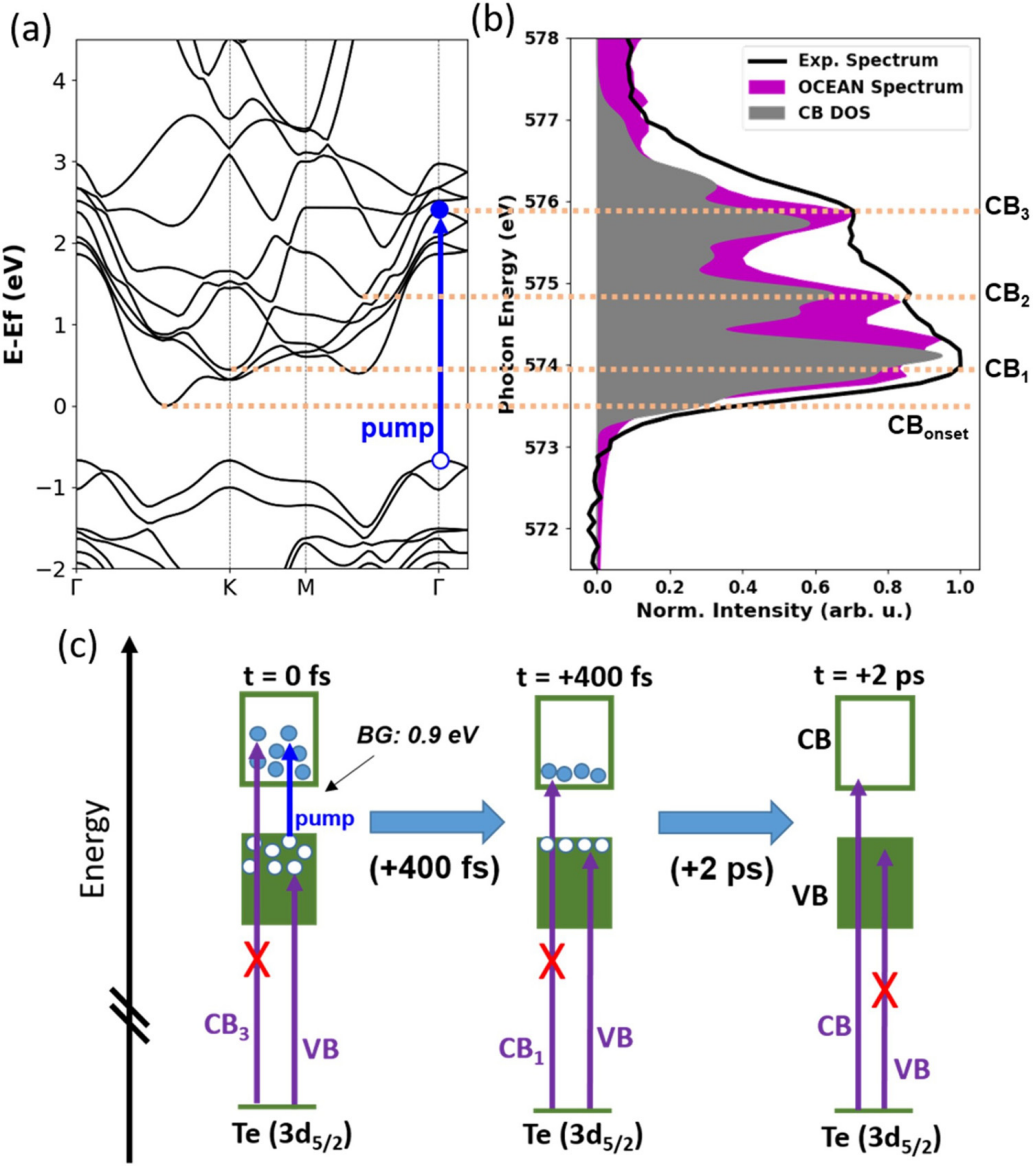
(a) Band structure of 2H-MoTe_2_ along the Γ−Κ−Μ−Γ path. One representative VB → CB transition induced by the 400 nm pump pulse is shown as a blue arrow. (b) Experimental static XAS, CB DOS, and OCEAN-simulated spectra are plotted and horizontal dashed lines are drawn at representative energies within the CB, labeled CB_1_, CB_2_, and CB_3_. (c) A model of the probing scheme by x-ray transient absorption of 2H-MoTe_2_. The VB and CB are shown schematically as filled and empty boxes and photoexcited electrons and holes are drawn as filled or empty circles, respectively. Representative x-ray transitions from the Te 3*d*_5/2_ core level are shown with and without an X to represent a decrease or increase in absorption relative to the static spectrum, respectively, due to state filling.

In Fig. 3(a), the DFT-calculated band structure of 2H-MoTe_2_ along the Γ−Κ−Μ−Γ path is plotted. In Fig. 3(b), the corresponding total CB DOS in 2H-MoTe_2_ is compared to the experimental absorption spectrum of the Te M_5_ pre-edge, after applying a global energy shift to the CB DOS to match the onset of the experimental absorption spectrum. In addition, BSE calculations of the Te M_5_ pre-edge transitions are performed using the OCEAN software package.^39,40^ The resulting OCEAN-simulated spectrum is plotted in Fig. 3(b), after applying a global shift to the energy scale to match the onset of the experimental absorption spectrum. The experimental M_5_ pre-edge spectrum has three major peaks, labeled CB_1_, CB_2_, and CB_3_ for convenience, and these peak energies match the distribution in the CB DOS and the OCEAN-simulated spectrum. The agreement between the energy of the critical points in the CB DOS and the Te M_5_ pre-edge absorption indicates that the Te 3d_5/2_ core-hole is well screened and the M_5_ XAS can be considered as a map of the CB unoccupied DOS in the valence shell. Therefore, changes in occupation of the CB at a particular energy within the band structure can be gauged by corresponding changes in the Te 3d_5/2_ → CB absorption via the state-filling description.

As shown in the projected/partial DOS (PDOS) plotted in Fig. S4 of the supplementary material, the VB also contains significant Te character, allowing for the state-filling picture to be used to describe the Te 3*d*_5/2_ → VB hole signal observed following optical excitation as well. The contribution of Mo character in the DOS implies that the Mo M_3,2_ edge could also be used, in principle, to map changes in VB/CB state occupation. However, as seen in Figs. S5 and S6 of the supplementary material, the Mo M_3,2_ edge is quite broad, leading to a mostly featureless pre-edge spectrum. The Mo M_3,2_ edge is also weak compared to the overlapping N K edge, which hinders the measurement in this region due to the use of Si_3_N_4_ as a substrate as well as a target for the I_0_ measurement.

### Capturing changes in the electronic structure with x-ray transient absorption

In the femtosecond time-resolved experiments performed at the PAL-XFEL, we photoexcite 2H-MoTe_2_ with a sub-100 fs, 400 nm pulse and measure the time-delayed XAS of the excited sample. The probing scheme is schematically depicted in Fig. 3(c). In the leftmost panel labeled t = 0 fs, the above-bandgap pump excitation (hν_400 nm_ = 3.1 eV compared with the bandgap of ∼0.9 eV) produces nonthermal excited carriers with an energy distribution centered significantly above the CB minimum (electrons) and below the VB maximum (holes). The state-filling signal is expected to map this distribution: the reduced Te 3d_5/2_ → CB absorption due to the excited electrons will initially have a dominant contribution at higher energies in the CB. Over the course of 10's–100's of femtoseconds following initial excitation, the carriers are expected to thermalize and cool due to known carrier–carrier and carrier–phonon scattering processes,^1,12^ as depicted in the schematic of Fig. 3(c), from the left panel to the middle panel. At these intermediate delay times following the thermalization/cooling process, the state-filling signal is expected to contribute closer to the Te 3*d*_5/2_ → CB minimum in the case of the electrons. The 3.1 eV (400 nm) pump excitation is chosen to produce hot electrons with a large energy difference between the initial hot electron signal (i.e., near Te 3*d*_5/2_ → CB_3_) vs the cooled electron signal (Te 3*d*_5/2_ → CB_1_). Finally, electron–hole recombination will lead to the decay of the state-filling signal, as depicted in the rightmost panel of Fig. 3(c). In addition to the state-filling effects, lattice heating via carrier-phonon scattering and nonradiative recombination can affect the core-level absorption edge. [Note that this is not shown in the schematic in Fig. 3(c)] Lattice heating often manifests as a broadening/shifting of the core-level edge jump in absorption compared to room-temperature XAS, leading to a long-lived transient feature near the onset of the core → CB edge (see the supplementary material for more details).^6,7,10,17^

### Femtosecond transient x-ray spectroscopy of 2H-MoTe_2_

**FIG. 4.**
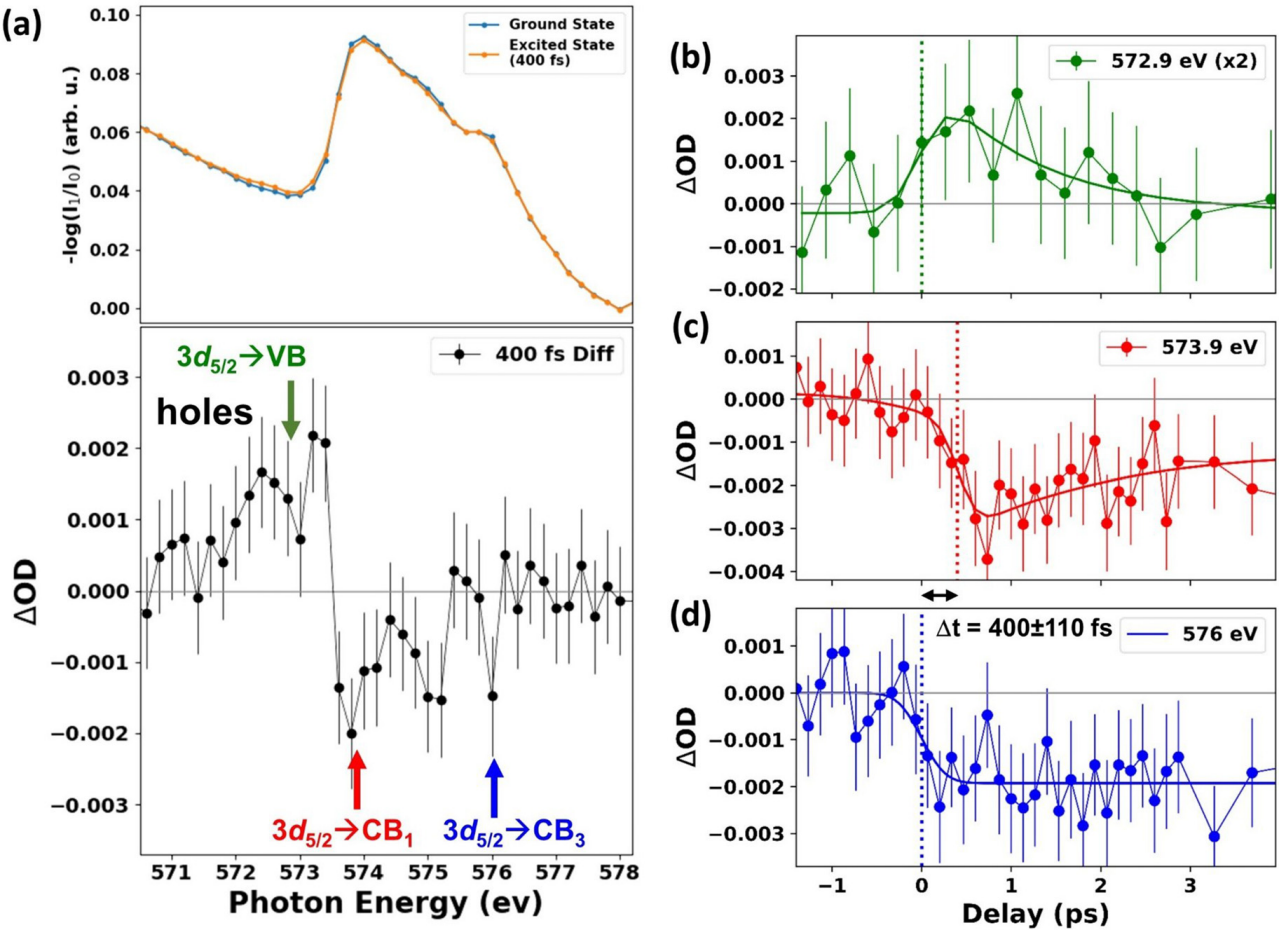
(a) Comparison between the Te M_5_ pre-edge absorption of 2H-MoTe_2_ in its ground state and ∼400 fs after 400 nm excitation (upper panel). The difference between ground- and excited-state spectra is shown in black circle and line (bottom panel). The error bars correspond to one standard deviation of the −log(I_1_/I_0_) measurements of all XFEL pulses for each energy. This differential absorption trace is the average of ∼10 h of accumulated data. (b)–(d) ΔOD (solid colored circles) as a function of time delay measured at the energies indicated by matching colored arrows in (a). The time-delay step size is 266 fs in (b) and 133 fs in (c) and (d). The full lines are fits corresponding to Gaussian-broadened step functions to describe the onset of all three features and additionally convolved with an exponential decay term in (b) and (c). The vertical dashed colored lines mark the time-delay center of the Gaussian-broadened step function for each feature. A typical delay scan collected at each monochromatized x-ray photon energy setting is an average of ∼1 h of accumulated data. Delay scans require shorter acquisition times compared to energy scans due to the 10 s wait time required to change monochromator energy setting, resulting in a significantly lower duty cycle for energy scans.

In Fig. 4(a), the Te M_5_-edge absorbance of 2H-MoTe_2_ measured at PAL-XFEL without photoexcitation is plotted (ground state, blue line) and at a delay time of ∼400 fs after optical excitation (excited state, orange line). The excited-state spectrum is an average of two transient spectra measured at nominal delay times of +300 fs and +500 fs (relative to the independent determination of time-zero described in the Experimental Methods and Calculations section). In the lower panel of Fig. 4(a), the differential absorption spectrum at 400 fs delay, defined as $\Delta OD\;(E,\tau )={OD}_{400\;\mathrm{fs}}(E,\tau )-{OD}_{\mathrm{static}}(E)$, is plotted. This differential spectrum consists of three main features. First, an increased absorption at 572.5 eV, approximately 1 eV below the 3*d*_5/2_ → CB onset. Second, a derivative feature centered at the 3*d*_5/2_ → CB edge onset, with increased absorption just below the edge and decreased absorption just above the edge. Finally, it also shows a general decrease in the absorption at energies above the 3d_5/2_ → CB edge (between 574 eV and 576 eV). The observed changes in the absorption amplitude are about ∼7% of the M_5_ pre-edge feature (static 3*d*_5/2_ → CB absorption). Thus, the expected optically induced transient changes in the transmission are approximately 0.14% (i.e., 7% of the 2% change in transmission in the static pre-edge absorption). Due to the small absorption edge jump at the Te M_5_ pre-edge, the difference spectrum in Fig. 4(a) required averaging over 10 h of data.

The features observed in the difference spectrum at ∼400 fs delay time are interpreted in terms of the middle panel of the schematic shown in Fig. 3(c). The increased absorption at 572.5 eV in the transient spectrum, ∼1 eV below the 3*d*_5/2_ → CB edge, is assigned to the photoexcited holes in the valence band. The derivative feature centered at 573.5 eV as well as the decrease in absorption above 574 eV are caused by a combination of bandgap renormalization (red-shifting of the edge), broadening due to the presence of free carriers and lattice-heat effects, and state filling by the electrons in the CB.^6,10,17,47^

In Figs. 4(b)–4(d), the ΔΟD measured at three chosen energies indicated by arrows in Fig. 4(a) are plotted as a function of time delay. The three chosen energies are (i) 572.9 eV, which corresponds to the 3*d*_5/2_ → VB hole signal close to the VB maximum, (ii) 573.9 eV, which corresponds to the 3*d*_5/2_ → CB_1_ signal close to the bottom of the CB (near the Κ and Μ critical points), and (iii) 576 eV, which corresponds to the 3*d*_5/2_ → CB_3_ signal involving higher-energy states within the CB (near the Γ critical point). The features at 572.9 eV (3*d*_5/2_ → VB) and 576 eV (3*d*_5/2_ → CB_3_) both appear to rise within the pump excitation at time-zero, whereas the feature at 573.9 eV (3*d*_5/2_ → CB_1_) appears to be slightly delayed. The rise of the 576 eV signal is fit to a convolution between a Gaussian function and a step function and the instrument-response function (IRF) is determined to be ∼200 fs, defined by the full width at half maximum (FWHM) of the Gaussian function. The time traces of the signals at 572.9 eV and 573.9 eV are each fit to a Gaussian broadened step function convolved with an exponential decay function. The onset of the 573.9 eV feature, defined by the center of the Gaussian broadened step function, t_0_, is extracted as t_0_ = 400 ± 90 fs and this is compared to the fitted onset of the 576 eV signal at t_0_ = 0 ± 70 fs. The uncertainties in t_0_ are the standard errors of the extracted best-fit parameter. The total delay in the onset of the 573.9 eV feature is therefore Δt = 400 ± 110 fs with respect to the fitted onset of the 576 eV signal, with the corresponding uncertainty determined after error propagation.

The appearance of the 3*d*_5/2_ → VB and 3*d*_5/2_ → CB_3_ signals at time-zero, within the IRF of the measurement, is consistent with a state-filling description where a distribution of carriers is produced immediately with the pump excitation. The optical excitation opens new transitions from the Te 3*d*_5/2_ core level to the transient holes in the VB (3*d*_5/2_ → VB) and leads to a state-filling signal by the photoexcited electrons reaching energies up to 2.2 eV above the CB minimum (3*d*_5/2_ → CB_3_). The Δt = 400 ± 110 fs delay in the rise of the negative ΔOD feature near the band edge at 573.9 eV (3*d*_5/2_ → CB_1_), on the other hand, is attributed to the time required for the electrons to cool to the bottom of the CB by intra- and intervalley scattering through phonon emission, as depicted schematically in Fig. 3(c) (left to middle panels). The electron–phonon scattering leads to an electron distribution closer to the CB minimum and to a delayed state-filling response at these 3*d*_5/2_ → CB_1_ energies, which is expected on the few-hundred femtosecond timescale.^1,12,48^ The measured electron cooling time of 400 ± 110 fs assigned here for 2H-MoTe_2_, following above-bandgap photoexcitation, is comparable to the hole cooling time of 380 ± 90 fs measured in the same material by XUV transient absorption.^12^ The 400 ± 110 fs delay measured in the present work between state-filling signals at CB_1_ compared to CB_3_, which we attribute here to electron cooling, was not visible in the XUV measurements due to overlapping signals of the Te 4d_5/2_ and 4d_3/2_ core levels. The 1.5 eV spin–orbit splitting of the Te N_5,4_ edges in the XUV prevents the distinction between CB_3_ and CB_1_, even in the static absorption measurement. We note that the 400 nm excitation in the present work produces carriers with considerably greater above-bandgap energy per photon compared to the broadband visible excitation (550–950 nm) used in the XUV experiment.

After the initial rise of the transients observed in Figs. 4(b)–4(d), the 3*d*_5/2_ → CB_3_ feature at 576 eV remains constant up to a delay time of 4 ps, whereas the time traces of the 3*d*_5/2_ → VB and 3*d*_5/2_ → CB_1_ features are characterized by complete or partial decay on this timescale. The decay component of these two kinetic traces [Figs. 4(b) and 4(c)] is fit to a single exponential function (yt=y0+Aexp−tτ, where y_0_ and A are arbitrary constants) and the extracted time constant, τ, is found to be τ_hole_ = 1.2 ± 0.6 ps for the 3*d*_5/2_ → VB hole signal and τ_electron_ = 1.2 ± 0.8 ps for the 3*d*_5/2_ → CB_1_ feature. These observations are consistent with electron–hole recombination and trapping, which occur on these timescales, i.e., leading to decay of the transient holes in the VB and of the photoexcited electrons in the CB. Although the carrier dynamics are expected to be dominated by an Auger recombination process,^49^ the exponential decay fitting is chosen to compare to exponential time constants extracted from recent THz and visible pump–probe studies on similar defect-rich 2H-MoTe_2_ samples.^27,28^ Our measured electron and hole lifetimes (τ_hole_ and τ_electron_) are consistent, within the error bars, with the carrier-averaged lifetimes extracted from the THz and visible pump–probe studies and the hole lifetime measured by XUV transient absorption.^12^

In addition to the consideration of state-filling effects, carrier-phonon scattering leads to lattice heating, which can also affect the 3*d*_5/2_ → CB absorption of 2H-MoTe_2_, even in the absence of photoexcited carriers.^6,7,10,17^ The observation of a long-lived, negative plateau in the 3*d*_5/2_ → CB ΔΟD signals at 573.9 eV and 576 eV after ∼1 ps is associated with these lattice heating effects, which dominate after carrier cooling and electron–hole recombination. To corroborate this assignment, the analogous observation of this effect was made at the Te N_5_ edge (Te 4*d*_5/2_ → CB) of 2H-MoTe_2_ using XUV transient absorption following both 800 nm and 400 nm photoexcitation (see the supplementary material for details).^12^ Similar long-lived, heat-induced effects are common in transient core-level absorption experiments of semiconductors.^6,7,9^

## CONCLUSIONS AND OUTLOOK

We measured ultrafast soft x-ray absorption spectra of polycrystalline 2H-MoTe_2_ thin films following 400 nm excitation using an XFEL at PAL. The differential absorption spectrum at a delay time of 400 fs provides a direct observation of photoexcited holes in the VB, which are identified by short-lived Te 3*d*_5/2_ → VB absorption transitions appearing ∼1 eV below the 3*d*_5/2_ → CB absorption edge. The transient spectrum is also characterized by a negative ΔΟD signal at the 3*d*_5/2_ → CB energies due to excited electrons in the CB, bandgap renormalization, and lattice-heating effects. The time traces of the Te 3*d*_5/2_ → VB and Te 3*d*_5/2_ → CB_1_ state-filling signals reveal hole and electron lifetimes of 1.2 ± 0.6 ps and 1.2 ± 0.8 ps, respectively, which is consistent with electron–hole recombination occurring on this timescale. These carrier lifetimes are also consistent with THz and optical pump–probe measurements, which previously provided carrier-averaged lifetimes.^27,28^ Furthermore, prior to electron–hole recombination, we observe a 400 ± 110 fs delay of the 3*d*_5/2_ → CB_1_ state-filling depletion involving states near the bottom of the CB compared to the 3*d*_5/2_ → CB_3_ depletion involving higher-energy states within the CB. We interpret this finding as a direct signature of hot electron relaxation within the CB due to intra- and intervalley scattering by electron–phonon interactions. The observation of electron cooling dynamics in the present work provides additional and complementary information to the recent XUV transient absorption study of 2H-MoTe_2_,^12^ which did not distinguish these electron relaxation dynamics due to overlapping spectral features of the neighboring spin–orbit split N_5_ and N_4_ edges.

HHG-based XUV transient absorption spectroscopy has a number of unique capabilities including: (1) demonstrated attosecond temporal resolution in the condensed phase,^50^ (2) simultaneous absorption at multiple elemental edges in a single measurement,^12^ and (3) intrinsic pump–probe delay stability due to the single amplified laser source for the pump and probe. The results presented here provide a benchmark for femtosecond transient absorption spectroscopy of semiconductor materials using the advancing capabilities of XFELs that operate at higher energies in the soft x-ray regime. Some of the distinct, promising, yet complementary capabilities offered by XFELs include: (1) access to higher-energy core edges with large spin–orbit splittings (>10 eV), which was exploited in the present work to minimize the adverse overlap of adjacent core-level absorption, (2) much higher photon flux per pulse, allowing for increased sensitivity via pulse-to-pulse normalization, surface sensitivity via TEY measurements,^51^ and the possibility to detect weak transient signals due to the reduction of the shot noise level, (3) smaller focal spot sizes (∼1–10 *μ*m),^15,16^ and longer absorption lengths at higher-energy x-rays, enabling future study of small semiconductor samples or individual domains of polycrystalline samples and thicker samples, respectively, and (4) greater tunability over the soft, tender, and hard x-ray energies for accessing K, L, M, etc., edges of elements, which opens the possibility of probing different orbital symmetries of the valence shell due to dipole selection rules. High-repetition rate XFELs, such as the European XFEL and LCLS-II (currently under construction) will further advance the sensitivity of this approach.

## AUTHORS' CONTRIBUTIONS

A.B. and A.R.A. contributed equally to this work.

## SUPPLEMENTARY MATERIAL

See the supplementary material for details on the sample preparation and characterization, excited carrier density estimation, PAL-XFEL energy calibration, partial/projected density of states calculations, static Mo M3-edge measurements, and XUV transient absorption results with 400 nm pump-long lived signals.

## Data Availability

The data that support the findings of this study are available from the corresponding author upon reasonable request.
